# Dipeptide repeat proteins are present in the p62 positive inclusions in patients with frontotemporal lobar degeneration and motor neurone disease associated with expansions in *C9ORF72*

**DOI:** 10.1186/2051-5960-1-68

**Published:** 2013-10-14

**Authors:** David MA Mann, Sara Rollinson, Andrew Robinson, Janis Bennion Callister, Jennifer C Thompson, Julie S Snowden, Tania Gendron, Leonard Petrucelli, Masami Masuda-Suzukake, Masato Hasegawa, Yvonne Davidson, Stuart Pickering-Brown

**Affiliations:** 1 Clinical and Cognitive Sciences Research Group, Institute of Brain, Behaviour and Mental Health, Faculty of Medical and Human Sciences, University of Manchester, Salford Royal Hospital, Salford M6 8HD, UK; 2 Clinical and Cognitive Sciences Research Group, Institute of Brain, Behaviour and Mental Health, Faculty of Medical and Human Sciences, University of Manchester, A V Hill Building, Oxford Rd, Manchester M13 9PL, UK; 3 Mayo Clinic, Jacksonville, FL FL32224, USA; 4 Department of Neuropathology and Cell Biology, Tokyo Metropolitan Institute of Medical Science, 2-1-6 Kamikitazawa, Setagaya-ku, Tokyo 156-8506, Japan

**Keywords:** Frontotemporal lobar degeneration, C9ORF72, p62 inclusions, Dipeptide repeat proteins

## Abstract

**Background:**

Cases of Frontotemporal Lobar Degeneration (FTLD) and Motor Neurone Disease (MND) associated with expansions in *C9ORF72* gene are characterised pathologically by the presence of TDP-43 negative, but p62 positive, inclusions in granule cells of the cerebellum and in cells of dentate gyrus and area CA4 of the hippocampus.

**Results:**

We screened 84 cases of pathologically confirmed FTLD and 23 cases of MND for the presence of p62 positive inclusions in these three brain regions, and identified 13 positive cases of FTLD and 3 of MND. All cases demonstrated expansions in *C9ORF72* by Southern blotting where frozen tissues were available. The p62 positive inclusions in both cerebellum and hippocampus were immunostained by antibodies to dipeptide repeat proteins (DPR), poly Gly-Ala (poly-GA), poly Gly-Pro (poly-GP) and poly Gly-Arg (poly-GR), these arising from a putative non-ATG initiated (RAN) sense translation of the GGGGCC expansion. There was also some slight, but variable, immunostaining with poly-AP antibody implying some antisense translation might also occur, though the relative paucity of immunostaining could reflect poor antigen avidity on the part of the antisense antibodies. Of the FTLD cases with DPR, 6 showed TDP-43 type A and 6 had TDP-43 type B histology; one had FTLD-tau with the pathology of corticobasal degeneration. There were no qualitative or quantitative differences in the pattern of immunostaining with antibodies to DPR, or p62, proteins between TDP-43 type A and type B cases. Ratings for frequency of inclusions immunostained by these poly-GA, poly-GP and poly-GR antibodies broadly correlated with those for immunolabelled by p62 antibody in all three regions.

**Conclusion:**

We conclude that DPR are a major component of p62 positive inclusions in FTLD and MND.

## Background

Frontotemporal Lobar Degeneration (FTLD) is a clinical, pathological and genetically heterogeneous condition. The major clinical syndromes principally involve personality and behavioural change (behavioural variant frontotemporal dementia, or bvFTD) or language alterations of a fluent (semantic dementia) or non-fluent (progressive non-fluent aphasia) nature [1]. All three syndromes can be accompanied by Motor Neurone Disease (MND) though the combination of FTD and MND is most common. Histologically, around half of cases have tau-based pathology, half have TDP-43-based pathology, and about 5% have FUS-based pathology [2,3]. Importantly, around 40% of all cases have a strong family history of similar disease, irrespective of clinical or histological subtype [1].

By 2007, causative mutations had been identified in tau (*MAPT*) [4] and progranulin (*GRN*) [5,6]. Nonetheless, it was well known at that time that a number of large, independent FTD + MND kindreds demonstrated linkage to chromosome 9 in the region 9p21.2-p13.3 [7-10]. Subsequently, three GWAS studies for MND [11-13], and one for FTLD [14] identified a susceptibility locus within this linked region, with strongest association coming from a 80 kb haplotype block containing 3 genes, *MOBKL2B*, *IFNK* and *C9ORF72*. It has now been shown that this at least some of this association is due to the presence of a large hexanucleotide (GGGGCC) in *C9ORF72* gene in patients with both FTLD and MND [15,16]. The expansion occurs in the first intron or the promoter region of the gene, depending upon the transcript isoform in question, and can number up to as many as 1500 repeats. The expansion is found in about one in every twelve patients with FTLD, but this varies depending on geographical region with the expansion being rare in Asia [16].

Pathologically, most FTLD cases with the expansion [15-20], like many non-mutational cases of FTLD [2,21,22], show inclusion bodies within neurones (NCI) and glial cells of the cerebral cortex and hippocampus that contain the nuclear transcription factor, TDP-43, and are said bear a TDP-43 histological subtype termed FTLD-TDP type B (according to [23]), compatible with a clinical diagnosis of FTD and MND. Others, however, show a TDP-43 histological type characterised by the presence of many short neurites (DN) along with NCI within the outer layers of the cerebral cortex, and termed FTLD-TDP type A [23]. Nonetheless, irrespective of TDP-43 histological type, expansion carriers also show a unique pathology within the hippocampus [17] and cerebellum [17-19,24] characterised by NCI that are TDP-43 negative, but immunoreactive to p62 protein.

P62 is a marker for proteins destined for proteasomal degradation though the precise target protein within such NCI remains uncertain, and the pathogenetic mechanism underlying the hexanucleotide expansion remains unclear. Nonetheless, this is likely to result from one, or a combination, of three possible mechanisms: i) haploinsufficiency leading to loss of C9orf72 protein [15,16], ii) the expansion might form nuclear foci of toxic RNA and sequester other RNA-binding proteins such as Pur Alpha (Pur α) [25], or iii) RAN (repeat associated non ATG-initiated) translation of the expanded repeat region may lead to the 'inappropriate’ formation of dipeptide repeat proteins (DPR) which may aggregate and confer neurotoxicity [26,27].

Here, we show that DPR are at least one of the target protein(s) within the TDP-43 negative, p62-positive NCI in cases of FTLD associated with hexanucleotide (GGGGCC) expansions, and that such peptides are not associated with other histological forms, or genetic subtypes, of FTLD.

## Methods

### Patients

Brain tissues were available in the Manchester Brain Bank from a series of 84 patients with FTLD and 23 with MND. All patients were from the North West of England and North Wales. All FTLD cases fulfilled Lund-Manchester clinical diagnostic criteria for FTLD [28,29], and all those with MND fulfilled El Escorial criteria [30]. All brains had been obtained with full ethical permission following consent by the next of kin.

### Histological methods

Serial paraffin sections were cut (at a thickness of 6 μm) from formalin fixed blocks of temporal cortex (with hippocampus) and cerebellar cortex. Sections within the series were immunostained by routine methods for amyloid β protein (Aβ), tau, TDP-43 and FUS proteins, employing microwaving in 0.1 M citrate buffer, pH 6.0 for antigen retrieval [2]. Pathologically, of the 84 patients with FTLD, 30 had FTLD-tau (9 with FTLD-tau Picks (PiD), 7 with *MAPT* mutation, 11 with CBD and 3 with PSP), 52 had FTLD-TDP (24 with type A histology, 22 with type B histology, 6 with type C histology), 1 had FTLD-FUS (aFTLD-U) and 1 had FTLD-ni. (see [23] for definitions).

Further sections from each case within the series were screened for the presence of p62-immunoreactive NCI by immunostaining with p62-lck ligand (rabbit polyclonal antibody (B D Biosciences, Oxford, UK) 1:100) employing a standard ABC Elite kit (Vector, Burlingame, CA, USA) with DAB as chromagen, and again microwaving in 0.1 M citrate buffer, pH6.0 for antigen retrieval. Positive cases were defined where p62 positive, TDP-43 negative NCI within either the cerebellum (see [18]) or hippocampus (see [17]) could be clearly seen under low power objective (×20) and the majority of high power fields (×40) contained at least 2 NCI. Negative cases were either completely devoid of p62 immunostaining, or small amounts of apparently extracellular and 'extraneous’ p62 positive particulate material was observed in occasional high power fields.

### Dipeptide antibody staining

A non-ATG initiated translation of the expansion would putatively result in DPRs derived from either from forward (sense) (poly Gly-Pro, (poly-GP), poly Gly-Ala (poly-GA) and poly Gly-Arg, (poly-GR)) or reverse (antisense) (poly Ala-Pro (poly-AP), poly Pro-Gly (poly-PG) and poly Pro-Arg (poly-PR)) directions. Consequently, we commissioned a series of custom made rabbit polyclonal antibodies, raised against such putatively translated proteins. Briefly, peptides consisting of 15 repeats with an additional N terminal cysteine were synthesised and N-terminally conjugated to keyhole limpet haemocyanin prior to immunisation. All steps in the preparation were performed by Genentech. Antibodies were successfully raised against poly-GP, poly-GR, poly-AP and poly-PR proteins. However, it was not possible to generate antibodies to poly-GA and poly-PG proteins.

Serial sections from those cases showing p62-positive pathology, and those from 11 (non-p62 positive) cases with other histological and genetic forms of FTLD, other neurodegenerative disorders and healthy controls (see Table 1) were immunostained for DPR. Antibodies were employed in standard IHC (as above) though, in this instance, antigen unmasking was performed by pressure cooking in citrate buffer (pH 6, 10 mM) for 30 minutes, reaching 120 degrees Celsius and >15 kPa pressure Following titration to determine optimal immunostaining, antibodies were employed at dilutions of 1:500 (poly-AP and poly-PR), 1:750 (poly-GR) or 1:2000 (poly-GP).

**Table 1:** Selected clinical and pathological details of cases investigated by dipeptide immunostaining

***Case***	***M/F***	***Clinical diagnosis***	***Pathological diagnosis***	***Family history***	***Onset (y)***	***Duration (y)***	***Brain weight (g)***
1	M	FTD	FTLD-TDP type A	2 brothers, 2 sisters FTD	49	9	1050
2	M	FTD	FTLD-TDP type A	brother MND**, mother and grandmother FTD	60	8	1210
3	F	FTD	FTLD-TDP type A	none available	59	5	1140
4	M	FTD	FTLD-TDP type A	father dementia	64	8	1100
5	M	FTD	FTLD-TDP type A	father similar presentation, paternal grandmother 'AD’	63	2	na
6	M	FTD	FTLD-TDP type A	yes	78	4	1200
7	M	FTD + MND	FTLD-TDP type B	?paternal aunt said to be 'strange’	60	2	na
8	M	FTD + MND	FTLD-TDP type B	mother FTD	57	2	1210
9	M	FTD	FTLD-TDP type B	mother dementia	54	12	na
10	F	FTD	FTLD-TDP type B	mother and sister FTD	51	19	na
11	F	FTD + MND	FTLD-TDP type B	father 'AD’, sister MND, paternal nephew MND	63	2	na
12	F	FTD + MND	FTLD-TDP type B	sister MND, brother FTD, mother 'multiple sclerosis’	68	5	1363
13	M	FTD	Corticobasal degeneration	father and 5 sisters had Huntington’s disease	59	70	1271
14	M	MND	MND	brother FTD**, mother and grandmother FTD	60	5	1350
15	F	MND	MND	none available	40	5	1330
16	M	MND	MND	brother MND, sister FTD + MND	53	2	1250
17	M	FTD	FTLD-tau Pi	none	69	6	na
18	F	FTD	FTLD-tau *MAPT* +16	mother: early onset dementia; brother: MND	48	15	1100
19	M	Corticobasal Syndrome	FTLD-tau CBD	none	65	na	1020
20	M	FTD	FTLD-TDP A	mother AD, brother AD (but with behavioural problems)	66	7	980
21	F	FTD + MND	FTLD-TDP B	none available	50	2	1050
22	F	SD	FTLD-TDP C	none	70	2	1522
23	F	Alzheimer’s disease	Alzheimer’s disease	none	74	12	1220
24	M	Huntington’s disease	Huntington’s disease	none available	48	24	na
25	M	Huntington’s disease	Huntington’s disease	none available	56	19	1340
26	M	Normal	Normal control	none	54*	na	1720
27	F	Normal	Normal control	none	53*	na	1220

FTD = Frontotemporal dementia; MND = Motor Neurone Disease; SD = semantic dementia; FTLD = Frontotemporal Lobar Degeneration; na = data not available/applicable; * = age at death; ** = cases #2 and 14 were brothers.

Further sections from the series were immunostained with poly-GA (and poly-GP and poly-GR) antibodies courtesy of Dr M Hasegawa. These antibodies were raised against poly-(GA)_8_, poly-(GP)_8_ and poly-(GR)_8_ peptides with cysteine at N-terminus. The peptides were conjugated to *m*-maleimidobenzoyl-N-hydrosuccinimide ester-activated thyroglobulin. The thyroglobulin-peptide complex (200 μg) emulsified in Freund’s complete adjuvant was injected subcutaneously into a New Zealand White rabbit, followed by 4 weekly injections of peptide complex emulsified in Freund’s incomplete adjuvant, starting after 2 weeks after the first immunization. Immunoreactivities of these antisera were characterized by ELISA as follows. The peptide immunogens were coated onto microtiter plates. The plates were blocked with 10% fetal bovine serum (FBS) in PBS, incubated with the rabbit antisera diluted in 10% FBS/PBS at room temperature for 1.5 h, followed by incubation with HRP-goat anti-rabbit IgG (Bio-Rad) at 1:3000 dilution, and reacted with the substrate, 0.4 mg/mL *o*-phenylenediamine, in citrate phosphate buffer (24 mM citric acid, 51 mM Na_2_HPO_4_). The absorbance at 490 nm was measured using Plate Chameleon (HIDEX). All antibodies were used for imminohistochemistry at dilution of 1:2000, and pretreated as above. Sections were also immunostained with the anti-dipeptide repeat antibody C9RANT [26] (gift from L Petrucelli) at 1:3000 dilution.

### Microscopic analysis

Sections of cerebellum and hippocampus immunostained for p62 and each of our own 4 DPR antibodies, and the poly-GA antibody supplied by M Hasegawa were assessed for the presence of DPR immunostained NCI within granule cells of the cerebellum, and dentate gyrus cells and CA4 pyramidal cells of the hippocampus at ×20 magnification. The frequency of DPR-immunoreactive NCI was assessed according to:

0 = no immunostained NCI present in any field.

1 = very few immunostained NCI present, in some but not all fields.

2 = a moderate number of immunostained NCI present in each field.

3 = many immunostained NCI present affecting most cells in each field.

4 = very many immunostained NCI present, affecting nearly all cells in every field.

### Southern blotting

Frozen brain tissue for Southern blotting was available for most cases employed in the study. Southern blotting was performed using a PCR DIG labelled probe adjacent to the expansion. The PCR probe consisted of 851 bp amplicon using the following primers, forward 5′ CCCACACCTGCTCTTGCTA 3′; reverse 5′ CGTTCTGTGTGATTTTTAGTGATGA 3′.

Briefly, samples were digested overnight with 20 u of XbaI (New England Biolabs). Samples were electrophoresed in 0.8% agarose 1 xTBE gels run at 1.5 volts/cm for 18 hours. Following standard protocols [31], gDNA was transferred to positively charged nylon membrane. Membranes were fixed using UV light at 365 nm for 3 minutes using a GE Image quant 350. Membranes were hybridized and detected as per the DIG detection Manual (Roche Applied Science). Signals were visualised using the GE Image quant 350 after 1 to 4 hours.

### Expansion sizing and analysis

Expansion sizing was carried out using ImageQuant TL software (Version 7, GE Healthcare) sizing the repeat number against the DIG labelled lambda Hind III labelled size standard included on each gel (Roche Applied Science). Positive control (gDNA isolated from the B-Lymphocyte cell line ND06769 obtained from the NINDS Repository–Coriell) and negative control were included on each blot, and were required to show a band of the expected size or no signal on hybridisation respectively for each blot to pass quality control.

### Statistical analysis

Rating data from semiquantitative asssessments was entered into an excel spreadsheet and analysed using Statistical Package for Social Sciences (SPSS) software (version 17.0). Mann–Whitney test was used to compare rating data between pairs of groups. A p-value of less than 0.05 was considered statistically significant. Regression analysis using Intercooled Stata Version 9 (StataCorp) was carried to out to investigate effects between repeat length, age of onset and disease duration.

## Results

Screening the 84 FTLD and 23 MND cases with p62 revealed 16 cases, 13 with FTLD (cases #1-13) and 3 with MND (cases #14-16) (Table 1) showing p62 positive, TDP-43 negative NCI within granule cells of the cerebellum and other cerebellar cell types, and in granule cells of the dentate gyrus, and pyramidal cells of CA4, CA3 and CA2 regions of the hippocampus, as described previously [17-19,24]. Twelve of the 13 FTLD cases showed TDP-43 proteinopathy, classifiable [23] as either type A (6 cases) or type B (6 cases): the other case had tauopathy compatible with corticobasal degeneration (CBD) (Table 1). Eight of the FTLD cases had presented with bvFTD clinically, 4 with FTD + MND and one (with CBD pathology) with a combination of FTD and corticobasal syndrome. All MND cases bore typical TDP-43 pathological changes in motor neurones of brain stem nuclei and spinal cord (where this was available for analysis).

**Figure 1:**
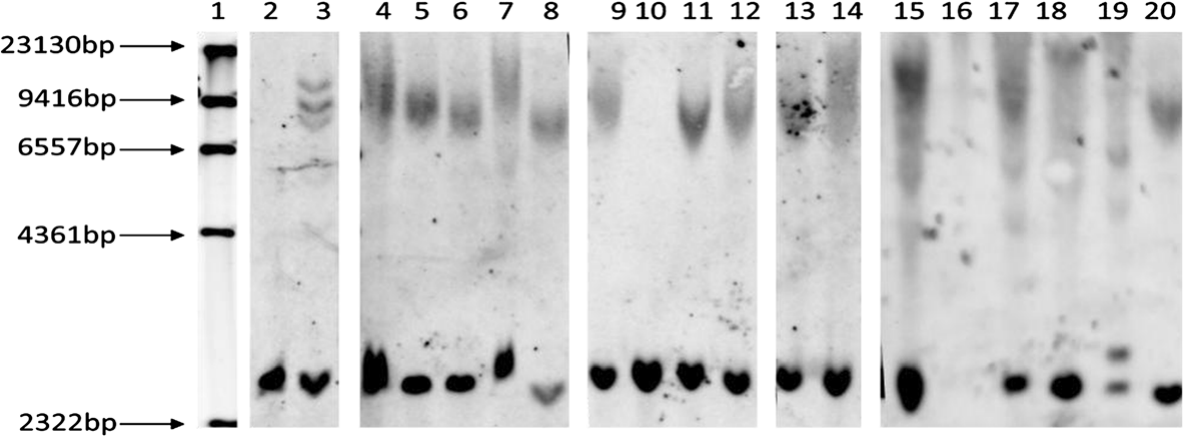
**Southern blotting of FTLD and MND cases bearing expansions in *C9ORF72*.** Lane 1: Marker, Lane 2: Negative control brain, Lane 3: ND06769, Lane 4: 01/06 FTD case #3, Lane 5: MND case #15, Lane 6: FTD/MND case #11, Lane 7: FTD case #10, Lane 8: CBD case #13, Lane 9: FTD case #1; Lane 10: control FTD case with tauopathy, Lane 11: FTD case #9, Lane 12: FTD/MND case #12, Lane 13: FTD case #2; Lane 14: MND case #14, Lane 15: FTD/MND case #7, Lane 20: MND case#16. Lanes 16-19 show expansions in other clinically diagnosed cases of MND (positive controls) where no brain tissue was available.

Frozen tissue was available for 12 of the 16 cases showing p62 pathological changes in cerebellum and hippocampus. These included 9 cases with FTLD (cases #1-3, 7, 9-13) and all 3 cases with MND (cases #14-16) (Table 1). All 12 of these cases demonstrated a pathological expansion in *C9ORF72* by Southern blotting. Expansion size ranged from ~5 kb (~450 repeats) to in excess of 23 kb (over 3600 repeats) (Figure 1). No expansions were detected in cases where no p62 pathological changes were observed. There was no correlation between repeat size and age of disease onset or duration (see Additional file 1: Figure S1).

### p62 immunostaining

**Figure 2:**
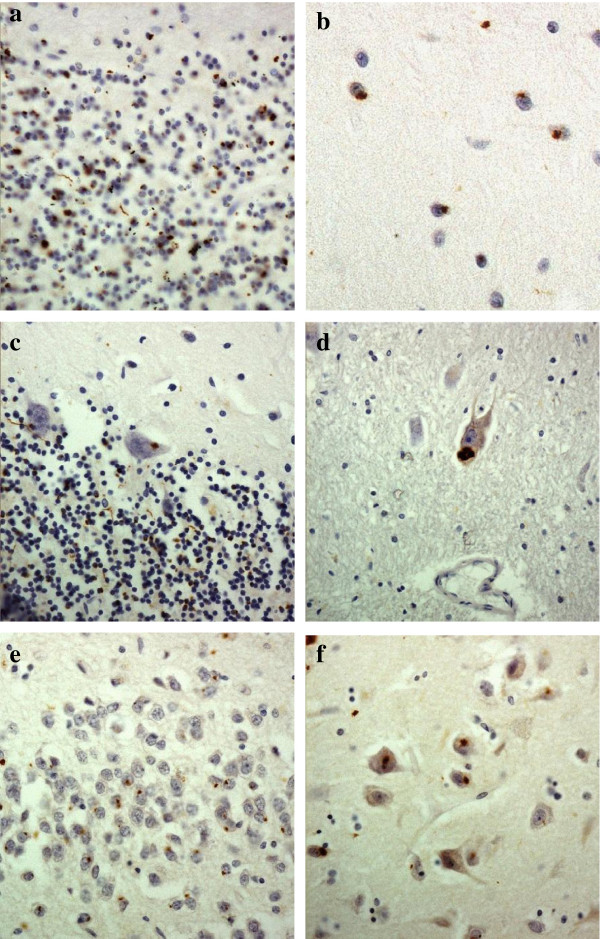
**Immunostaining of the cerebellum and hippocampus for p62 proteins shows neuronal cytoplasmic inclusions in granular neurones (a), basket cells (b), Purkinje cells (c) and cells of the dentate nucleus (d) of the cerebellum, and in dentate gyrus granule cells (e) and pyramidal cells of CA4 region (f).** Immunoperoxidase–haematoxylin. All × 40 microscope objective magnification.

In FTLD cases, on p62 immunostaining, NCI in granule cells of the cerebellum appeared as small rounded or oat-shaped bodies, though occasionally larger, more rounded and solid NCI were observed (Figure 2a). These were very abundant in case #1, common in cases #4, 7 and 10, moderately plentiful in cases #2 and 5, occasional in cases #3, 8 and 12, and rare in cases #6, 9 and 11 (Table 2). Similar, more granular, NCI were usually present in basket cells (Figure 2b), especially when granule cell inclusions were frequent, but none were seen within Golgi neurones, or within Bergmann glia. In some cases, occasional Purkinje cells (Figure 2c) and neurones in the dentate nucleus (Figure 2d) contained small, spicular or granular p62-immunoreactive structures, but these were not immunoreactive with TDP-43 antibodies. In most cases, occasional cells resembling astrocytes were seen to contain p62 immunoreactive intranuclear inclusions, these being located deep in the granule cell layer. All cases also showed abundant, small, rounded NCI within granule cells of the dentate gyrus (Figure 2e), along with spicular or granular inclusions within pyramidal cells of areas CA2/3 and CA4 (Figure 2f), less commonly in CA1 and subiculum (Table 2).

**Table 2:** **Ratings for p62 and DPR antibody immunostaining of neuronal cytoplasmic inclusions for all 15 cases with expansions in *C9ORF72***

**Case**	**CA4**						**DG**						**GCC**					
	**p62**	**poly-GA**	**poly-GP**	**poly-GR**	**poly-PR**	**poly-AP**	**p62**	**poly-GA**	**poly-GP**	**poly-GR**	**poly-PR**	**poly-AP**	**p62**	**poly-GA**	**poly-GP**	**poly-GR**	**poly-PR**	**poly-AP**
1*	2	2	3	3	0.5	0.5	1	2	0	0.5	0.5	0.5	4	4	3	0.5	0	0
2*	3	3	2	3	0.5	1	2	2	0.5	2	0.5	1	2	3	3	0	0	0
3*	3	3	3	2	0.5	0.5	4	3	3	0	0	0	1	4	2	2	0	0
4*	2	2	3	3	0.5	0	3	2	3	0	0.5	0	3	3	3	0	0	0
5*	2	1	3	3	0.5	0.5	3	3	2	0	0	0	2	3	3	1	0	0
6*	1	2	2	1	0	0	1	2	2	0	0	0	1	1	1	0	0	0
7**	3	3	3	3	0	0	3	3	2	0	0	0	4	4	4	0	0	0
8**	na	na	na	na	na	na	na	na	na	na	na	na	1	1	1	0	0	0
9**	3	2	3	3	0.5	0.5	1	2	1	0.5	0	0	0.5	3	1	0	0	0
10**	3	2	2	2	0.5	0.5	4	3	3	1	0.5	0	3	3	2	0	0	0
11**	3	3	3	3	0	0	4	3	2	2	0	1	0.5	3	2	1	0.5	0
12**	3	3	2	3	0	0	4	3	1	1	0	0	1	3	3	1	1	0
13	0	2	2	2	0	0	0	2	2	0	0	0	1	1	1	0	0	0
14	3	2	3	3	0	1	1	2	2	0	0	0	3	4	2	0	0	0
15	1	1	1	1	0	0	2	2	2	2	0	0	1	1	1	0	0	0
16	2	2	2	0	0	0	2	2	2	2	0	0	3	3	3	0	0	0

Cases #1-6 had FTLD TDP-43 type A histology (*), cases #7-12 had FTLD TDP-43 type B histology (**), case #13 had corticobasal degeneration, and cases #14-16 had Motor Neurone Disease.

CA4 = pyramidal cells of CA4 region of hippocampus; DG = dentate gyrus granule cells; GCC = granule cells of the cerebellum; na = no data.

In MND cases, cerebellar granule cell NCI were abundant in cases #14 and 16, but were rare in case #15 (Table 2). In all cases, p62 positive NCI were also observed within granule cells of the dentate gyrus, and similar (to FTLD cases) small, spicular or granular p62-immunoreactive inclusions were seen within pyramidal cells of areas CA2/3 and CA4, less commonly in CA1 and subiculum. Both of these kinds of changes were occasionally present in cases #14 and 15 but were moderately common in case #16 (Table 2).

In all FTLD and MND cases variable, but often many, pyramidal neurones, chiefly within the deeper layers of the adjoining cerebral (temporal) cortex also contained NCI similar to those in the hippocampus CA regions (not shown).

### DPR immunostaining

Results of immunostaining for DPR are shown in Table 2. All FTLD and MND cases showed similar patterns of immunostaining though there were quantitative differences between cases with respect to the number of inclusions immunostaining.

**Figure 3:**
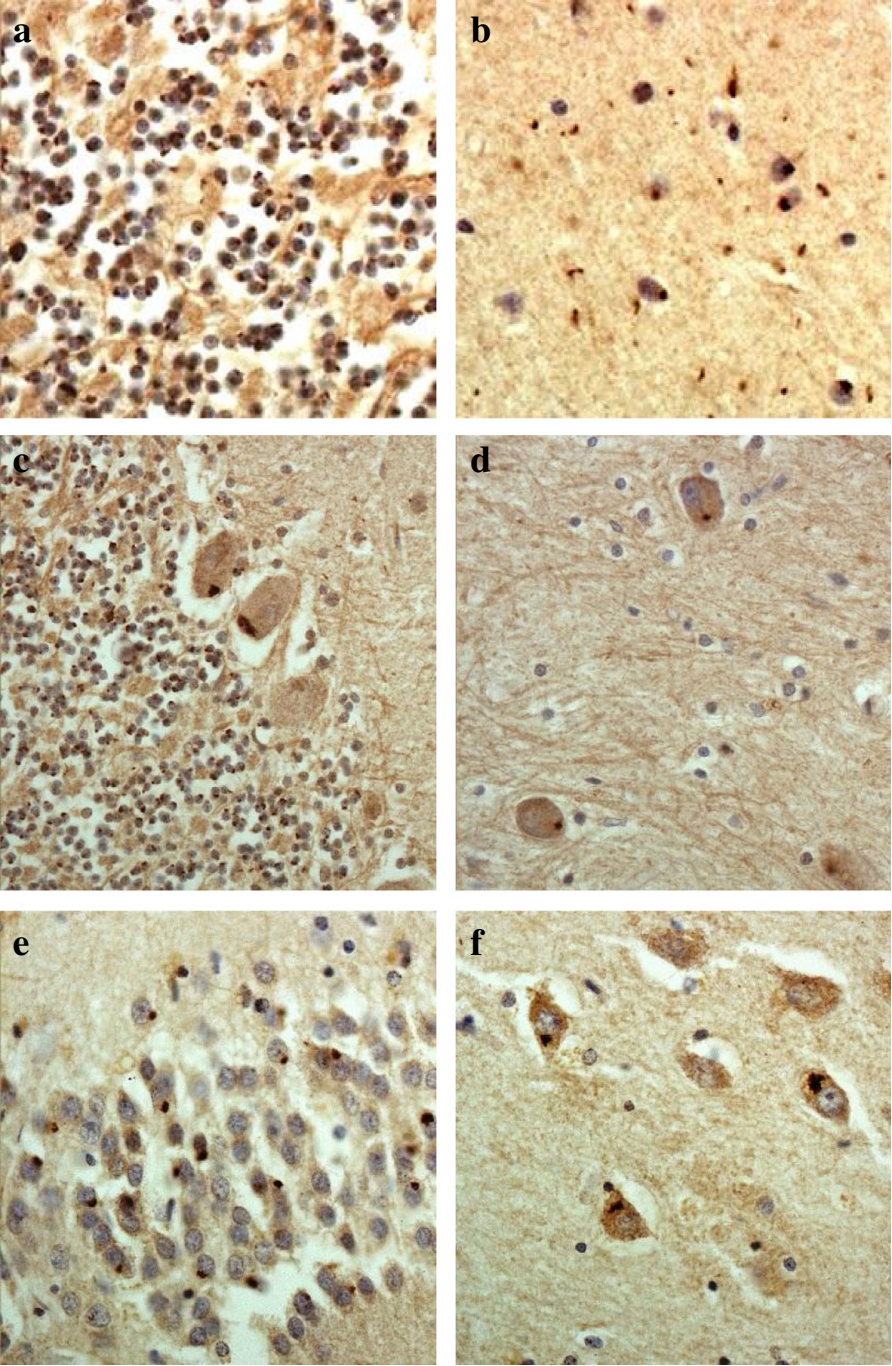
**Immunostaining of the cerebellum and hippocampus for poly-GA protein shows neuronal cytoplasmic inclusions in granular neurones (a), basket cells (b), Purkinje cells (c) and cells of the dentate nucleus (d) of the cerebellum, and in dentate gyrus granule cells (e) and pyramidal cells of CA4 region (f), similar to those seen on p62 immunostaining.** Immunoperoxidase–haematoxylin. All × 40 microscope objective magnification.

As with p62 immunostaining, immunostaining with both our own custom anti poly-GP and poly GR antibodies and those prepared by Hasegawa similarly detected NCI in granule cells of the cerebellum, as did Hasegawa’s poly-GA antibody (Figure 2a). Again, more granular looking NCI were usually present in basket cells (Figure 2b), occasionally in Purkinje cells (Figure 2c) and neurones in the dentate nucleus (Figure 2d), but none were seen within Golgi neurones, or within Bergmann glia. A punctate, or filamentous, staining was also seen within the molecular layer of the cerebellum, this probably relating to parallel projection fibres (Figure 3a and b). No nuclear inclusions in atrocytes immunostained with p62 appeared to be detected by poly-GA antibody. All FTLD and MND cases also showed abundant, small, rounded NCI within granule cells of the dentate gyrus (Figure 3e), along with spicular or granular inclusions within pyramidal cells of areas CA2/3 and CA4 (Figure 3f), but these were less commonly seen in CA1 and subiculum (Table 2).

**Figure 4:**
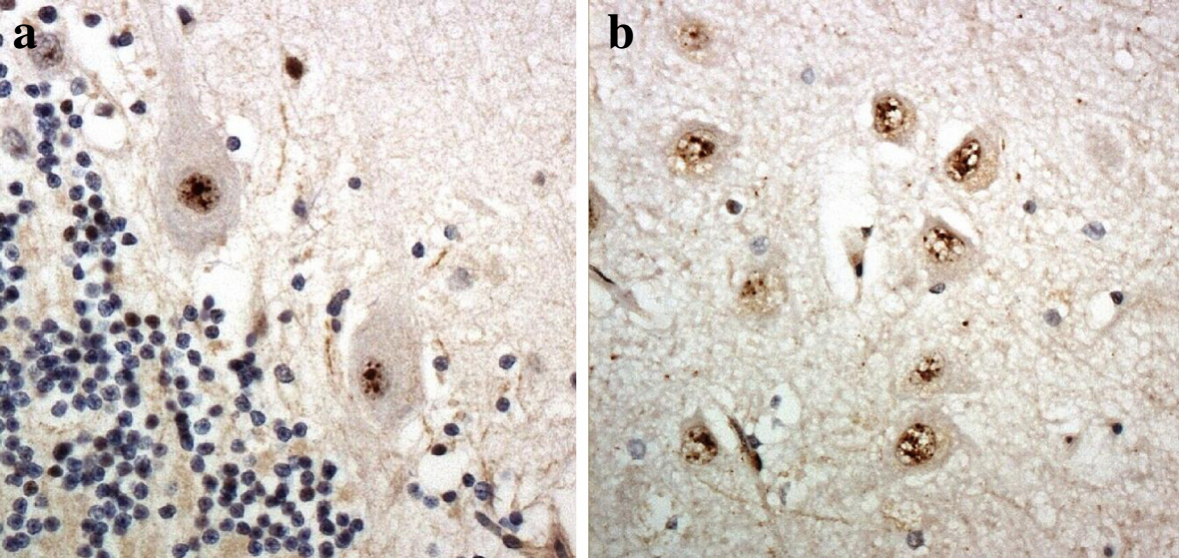
**Immunostaining of Pukinje cells of the cerebellum (a) and pyramidal cells of CA4 region of hippocampus (b) for poly-PR protein shows strong immunoreactivity of chromatin.** Note lack of staining of NCI in either cell type. Immunoperoxidase–haematoxylin. All × 40 microscope objective magnification.

Our own custom antibody raised against poly-AP showed rare, and weak, immunostaining of NCI within CA4 neurone in occasional cases were (Table 2), similar in appearance to those seen in such cells on p62 immunostaining, or with poly-GA, poly-GP and poly GR antibodies. The antibody to poly-PR did not stain NCI in any case, but strongly immunostained chromatin granules, especially in Purkinje cells of cerebellum and CA4 neurones of hippocampus in some cases bearing expansions in *C9ORF72* (Figure 4). However, not all expansion bearers showed any such immunostaining, nor did any of the control cases.

The pattern of immunolabelling of NCI with C9RANT antibody in cerebellum and hippocampus mirrored that reported by others [26], and again appeared to be identical to that seen with both our own poly GP antibody and that prepared by M Hasegawa (not shown).

In all expansion carriers, variable numbers of, but often many, pyramidal neurones within the deeper layers of the adjoining cerebral (temporal) cortex also contained NCI immunostaining with antibodies to DPR in a similar fashion to those in the hippocampus CA regions (not shown).

Cases of FTLD of other histological or genetic subtypes showed no immunostaining of TDP-43 positive NCI, DN or NII, or of tau positive structures (Pick bodies, neurofibrillary tangles). The non-demented control cases showed no relevant DPR immunostaining, and the NII within the HD cases were also unstained by any DPR antibody.

In all instances, immunostaining of NCI with anti-DPR antibodies was robust. There were no apparent effects of variable post mortem delay or prolonged fixation upon the quality or quantity of NCI immunostaining, with cases that had been stored in formalin for up to 20 years before new blocks had been taken for processing into wax sections still showing robust immunostaining for DPR, as did cases where post mortem delay periods had exceeded 4 days.

### Comparison of p62 and DPR immunostaining between FTLD-TDP subtypes

There were no statistically significant differences between ratings for p62 and DPR immunostaining in either FTLD-TDP type A or type B cases for NCI in granule cells of the cerebellum, or for those of the dentate gyrus, and CA4 neurones, of hippocampus.

### Comparisons and correlations between p62 and DPR immunostaining

The numbers of NCI immunostained by each DPR antibody did not always correspond either when compared to p62 immunostaining, or when compared among themselves. Those in CA2, CA3 and CA4 of hippocampus, and those in Purkinje cells and cells of the dentate nucleus appeared numerically similar in p62 and DPR immunostaining (except for poly-AP), though it appeared from microscopic inspection that p62-positive NCI in granule cells of the cerebellum were not all, or always, immunostained by both poly-GR and poly-GP antibodies, and none were stained by poly-AP antibody (Table 2). In the hippocampus only a minority of the p62-positive NCI in the dentate gyrus were immunostained by poly-GR and poly-GP antibodies, and again none were stained by poly AP antibody (Table 2).

Ratings for DPR immunostaining of NCI were correlated with those for each other, and those for p62 immunostaining, in granule cells of cerebellum, and dentate gyrus and CA4 neurones of hippocampus. In general, there were significant correlations between p62, poly-GA, poly GP and poly-GR ratings, but not with those involving poly-AP and poly-PR. Hence, ratings for poly-GA correlated significantly with those for p62 in cerebellum (p = 0.032), dentate gyrus (p = 0.000) and CA4 (p = 0.006), with poly-GR in CA4 region (p = 0.044) and with poly-GP in cerebellum (p = 0.003). In CA4, ratings for poly-GP correlated with those for poly-GR (p = 0.007). In the cerebellum, ratings for poly-GA correlated with those for poly-GP (p = 0.008), as did ones for poly-GR with poly-PR (p = 0.027). Only in CA4 did poly-AP correlate with poly-PR ratings (p = 0.014).

### Other observations

Cells containing DPR did not show obvious cell loss, or any other outward signs of neurodegeneration, whether these were granule cells of dentate gyrus or cerebellum, or pyramidal cells of CA regions or cerebral cortex, or Purkinje neurones of the cerebellum. No cases with *C9ORF72* expansion showed excessive tau and Aβ pathology (i.e. commensurate with a pathological diagnosis of AD) for age.

## Discussion

In the present study we have observed p62 positive, TDP-43 negative NCI within cerebellar granule cells, and granule cells of the dentate gyrus and pyramidal cells of the hippocampus (see [17-19,24]) in 13 of 84 (15%) cases of FTLD and in 3 of 23 (13%) cases of MND. Interestingly, all cases with expansion showed a FTD, FTD + MND or MND clinical phenotype, and 15/16 bore appropriate TDP-43 protein pathological changes. However, one expansion carrier with clinical FTD (case #13 in Table 1) was of FTLD-tau with CBD pathology. This case was described by us previously [20] and is important as it is only the second case of CBD to be described with expansion in *C9ORF72* (see [32] for details of the other case), though there was no post-mortem confirmation of CBD in this latter instance. The present case demonstrated an expansion in *C9ORF72* both on repeat-primed PCR (not shown), and on Southern blot (see lane #8 in Figure 1). Although the case showed no TDP-43 pathology, DPR were present in cells of the cerebellum and hippocampus (see Table 2), though curiously those NCI in granule cells of the cerebellum were p62-immunopositive, while those in hippocampus dentate gyrus and CA4 regions were not positive for p62.

None of the other cases with other histological or genetic forms of FTLD showed p62 positive, TDP-43 negative NCI within either cerebellar granule cells or within the hippocampus. The pathological findings described here are therefore broadly consistent with those previously reported by others in unselected series of cases of FTLD and/or MND [17-19,24].

All of the 12 p62 positive cases where frozen brain tissues were available for analysis showed an expansion in *C9ORF72* by Southern blotting, and are therefore consistent with the suggestion [17,18] that this type of p62 pathology is pathognomic for cases of FTLD and/or MND associated with *C9ORF72* mutation. Thus, it can be presumed that an expansion was also present in the other four FTLD cases with relevant p62 pathology where no southern blotting (or repeat primed PCR) was possible due to lack of fresh frozen brain tissue. The lack of association between expansion length and age of disease onset or duration presumably reflects that a minimum size of expansion is required for disease. Present data suggests this might be around 500 repeats, and expansions beyond this are not additionally detrimental.

The exact target protein within the p62 immunoreactive NCI remains uncertain. Here, we performed immunohistochemistry employing antibodies to DPR putatively produced in the brain through non-ATG initiated (RAN) translation of the expansion itself. Although double immunofluorescence labelling was not performed in the present study, microscopic observations on consecutive serial sections stained with each DPR antibody suggested that most, if not all, of the p62-positive NCI within Purkinje and dentate nucleus cells of the cerebellum, and neurones of CA2, CA3 and CA4 of the hippocampus are similarly immunoreactive to poly-GA, poly-GP and poly-GR antibodies (and to a much lesser extent, poly-AP antibody), whereas only a (variable) subset of p62-positive NCI within granule cells of the cerebellum and dentate gyrus appeared immunoreactive to these antibodies. These microscopic impressions were supported by statistical analysis showing good correlations between ratings of inclusion body frequencies as assessed using poly-GA, poly-GP and poly-GR antibodies. Other studies [26,27] where double immunofluorescence labelling was indeed performed substantiate the present observations.

None of the NCI was immunoreactive to poly-PR antibody, though this antibody did immunolabel chromatin granules, especially in Purkinje cells and CA4 neurones, in some *C9ORF72* expansion bearers. Although this kind of immunostaining was not seen in any of the control subjects in such cells, it was not consistently present in all expansion bearers, and so the relevance of this (to pathogenesis) remains uncertain. None of the DPR antibodies were immunoreactive to any other kinds of inclusions (TDP or tau) associated with other histological or genetic forms of FTLD, or the NII containing CAG repeats seen in HD. These findings are consistent with those recently reported by Mori and colleagues [27]. The pattern of DPR immunolabelling in the cerebellum and hippocampus in *C9ORF72* expansion bearers mirrored that reported by Ash et al. [26], and this appeared identical to that shown by our own poly GP antibody, and that prepared by M Hasegawa. C9RANT antibody was raised to a panel of dipeptide repeat immunogens [26], but judging from the similarities between its pattern of immunolabelling and that of our own poly-GP antibody, it might be considered that its specificity, or at least, it avidity is greatest for the poly-GP component of the immunogen mix. The finding of strong and consistent staining with poly-GA, poly-GP and poly-GR antibodies suggests that most of the aberrant translation relates to sense transcripts, though the slight, but variable immunostaining with poly-AP antibody implies some antisense translation might also occur. However, it remains possible that the relative paucity of immunostaining seen here with antibodies to antisense transcripts reflects poor antigen avidity on the part of the antibody rather than indicating an absence of the relevant polypeptide with DPR inclusions *per se*.

Nevertheless, it appears that DPR may not be the only proteins present within these structures since the p62 positive inclusions have also been shown to contain hnRNPA3 [33], and others reported the inclusions in granule cells of the dentate gyrus and cerebellum (at least) to be immunoreactive to ubiquilin-2 [34]. There is little or no DPR immunostaining of TDP-43 in hippocampus or cerebral cortex in either expansion bearers, or patients with other histological forms of FTLD [29], consistent with those observations of NCI based on p62 immunostaining [17-19,24]. Furthermore there was no immunostaining of NII containing expanded poly-Q stretches in patients with HD (see also [27]). Collectively, these findings reinforce the suggestion that p62-, DPR-positive NCI are pathognomic for FTLD (and MND) associated with *C9ORF72* expansions.

It remains uncertain as to how these NCI containing DPR may relate to disease pathogenesis and other fundamental aspects of disease pathology such as TDP-43 positive NCI and neurites. While NCI containing DPR are located in clinically and pathologically relevant regions, such as the hippocampus and adjacent temporal cortex, they are actually present in cells distant from the major TDP-43 changes (layers V and VI in cerebral cortex and areas CA2/3/4 in hippocampus), although it is acknowledged that cells in the dentate gyrus of the hippocampus may contain one or other, but rarely both, types of inclusion [19,27]. Those cells containing DPR did not show any other outward signs of neurodegeneration, whether these being small granule cells of dentate gyrus or cerebellum, or larger pyramidal cells of hippocampal CA regions, cerebral cortex or Purkinje neurones of the cerebellum. Likewise, there appeared to be no obvious loss (thinning out) of granule cells in either region or depletion of cells from hippocampal pyramidal or cerebellar Purkinje cell layers. Moreover, such changes in the cerebellum appear to be without functional (clinical) consequence, although there have been reports of cerebellar atrophy in expansion bearers [35,36].

Interestingly, there appeared to be no qualitative or quantitative differences between either the number, or the pattern, of DPR stained NCI between FTLD-TDP type A and type B cases, in either hippocampus or cerebellum. This observation begs the question as to the role of *C9ORF72* expansions in determining histological phenotype and their relationship to TDP-43 pathology. It is possible that variability in repeat size may have a role in this, but we were unable to show any obvious differences in repeat size between Type A and type B cases by Southern blot. Alternatively, it is possible that the expansion confers an 'additive’ effect, through induction of a process marked by p62 pathology, which is superimposed upon a 'background TDP-43 proteinopathy derived through a similar mechanism to that seen in non-expansion bearers sharing the same TDP-43 histological phenotype. If that were so, then it might be presumed that patients bearing the mutation suffer a 'double whammy’, and that the expansion plays no real part in determining the TDP-43 proteinopathy and the basic underlying disorder. However, if this 'chance scenario’ were true, then it might be anticipated that *C9ORF72* repeat expansions might commonly occur in association with other disorders, though there is little, and conflicting, evidence for this in, for example, Alzheimer’s disease [37-43] or Parkinson’s disease and other parkinsonian syndromes such as Corticobasal syndrome [44-47]. Indeed, it is possible that in at least some of these instances where an expansion has been reported in patients with conditions other than FTLD or MND, the underlying condition may still be FTLD, though presenting in an atypical way [39].

## Conclusion

Although it is clear that production of DPR from unconventional translation of the expansion is a feature of *C9ORF72* associated diseases, it is far from certain as to whether such changes drive cell damage and loss, and how they might relate to changes in TDP-43 function and contribute clinical disability. It remains an open question whether DPR mediated changes are anything more than a pathological 'curiosity’, but nonetheless they appear to provide a specific diagnostic tissue marker for the presence of the genetic expansion.

## Competing interests

The authors declare that they have no competing interests.

## Authors’ contributions

DMAM was responsible for study design, microscopic assessments, data analysis and paper writing. SR, JBC and SPB performed all genetic analyses and Southern blotting, and assisted with preparation of the manuscript. AR provided technical assistance. JCT performed statistical analysis. JSS assisted with case diagnosis and classification. TG and LP provided C9RANT antibody and details of specificity. MMS and MH provided antibodies and details of specificity, and assisted with manuscript preparation. YD performed all immunohistochemical staining. All authors read and approved the final manuscript.

## Supplementary Material

Additional file 1: Figure S1 Relationship between minimum and maximum size of repeat and age at onset/disease duration. Click here for file
