# Supplementary material for: Dipeptide repeat proteins are present in the p62 positive inclusions in patients with frontotemporal lobar degeneration and motor neurone disease associated with expansions in C9ORF72
Source: Acta Neuropathol Commun. 2013 Oct 14;1:68. doi: 10.1186/2051-5960-1-68 (PMC3893586; doi:10.1186/2051-5960-1-68)
Supplement: Additional file 1: Figure S1 — Relationship between minimum and maximum size of repeat and age at onset/disease duration. [file 2051-5960-1-68-S1.docx]

Xba 1 probe DATA

Minimum size of repeat and age of onset

 Regress P 0.663, R2 0.0139,

Minimum size of repeat and duration of disease

 Regress P = 0.284, R2 0.1136

Maximum size of repeat and age of onset

 regress 0.737, R2 0.0083

Maximum size of repeat and Duration

 P = 0.890, R2 0.0020
